# MicroRNAs in vascular tissue engineering and post-ischemic neovascularization^☆^

**DOI:** 10.1016/j.addr.2015.05.003

**Published:** 2015-07-01

**Authors:** Massimo Caputo, Jaimy Saif, Cha Rajakaruna, Marcus Brooks, Gianni D. Angelini, Costanza Emanueli

**Affiliations:** a Bristol Heart Institute, School of Clinical Sciences, University of Bristol, Bristol, UK; b RUSH University Medical Center, Chicago, IL, USA; c University Hospital Bristol NHS Trust-Vascular Surgery Unit, Bristol, UK; d National Heart and Lung Institute, Imperial College London, London, England, UK

**Keywords:** BM, bone marrow, CAD, coronary artery disease, CHD, congenital heart disease, CLI, critical limb ischemia, EC, endothelial cell, EPCs, endothelial progenitor cells, ECM, extracellular matrix, ESC, embryonic stem cell, GF, growth factor, IHD, ischemic heart disease, miR, microRNA, MI, myocardial infarction, MNC, mononuclear cell, NP, nanoparticle, PAC, proangiogenic circulating cell, PAD, peripheral arterial disease, MMP, matrix metalloproteinase, VSMC, vascular smooth muscle cell, TE, tissue engineering, VTE, vascular tissue engineering, MicroRNAs, Vascular tissue engineering, Therapeutic angiogenesis, Congenital/acquired heart disease, Aneurysms, Ischemic disease

## Abstract

Increasing numbers of paediatric patients with congenital heart defects are surviving to adulthood, albeit with continuing clinical needs. Hence, there is still scope for revolutionary new strategies to correct vascular anatomical defects. Adult patients are also surviving longer with the adverse consequences of ischemic vascular disease, especially after acute coronary syndromes brought on by plaque erosion and rupture. Vascular tissue engineering and therapeutic angiogenesis provide new hope for these patients. Both approaches have shown promise in laboratory studies, but have not yet been able to deliver clear evidence of clinical success. More research into biomaterials, molecular medicine and cell and molecular therapies is necessary. This review article focuses on the new opportunities offered by targeting microRNAs for the improved production and greater empowerment of vascular cells for use in vascular tissue engineering or for increasing blood perfusion of ischemic tissues by amplifying the resident microvascular network.

## Graphical abstract

Schematic of application of miRs in vascular tissue engineering. Cells from patient could be collected, expanded and be subjected to miR modulation strategies to generate cells of desired specification. Pluripotent stem cells can be modulated to inhibit self-renewal and promote differentiation toward a desired progenitor cell. Also miR modulation of the progenitor cells may permit maintenance of the cells in a progenitor state or differentiation toward specific progeny thereby facilitating a modular cell therapy strategy. The modified cells can be loaded onto scaffolds for implantation or can be used to generate intact tissues and organs *in vitro* before implantation in the patient. MiR modification strategies can also be used directly in ischemic tissues to regulate angiogenesis. Extracellular vesicles (EVs) carrying the ‘desired’ cargo of miR can be isolated from stem or progenitor cells for direct injection into ischemic tissue.

**Figure:**
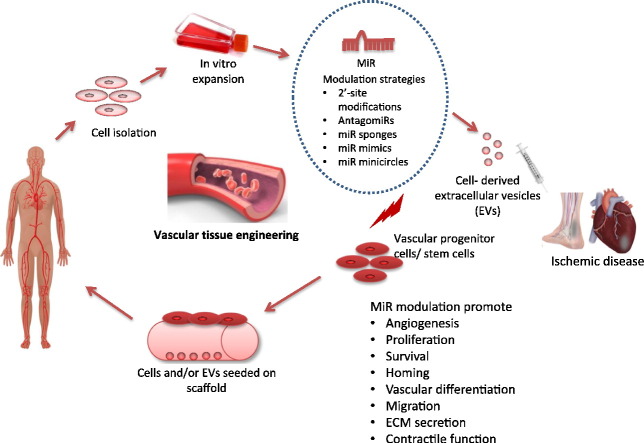

## 1 Introduction

The vasculature is one of the first organ systems to develop and it forms an extensive network throughout the body mediating gas exchange, transport of nutrients and waste products, as well as delivering cells and mediators involved in immunity. Blood vessels mainly consist of endothelial cells (ECs) that line the internal surface of the entire vascular system and mural cells, *i.e.* vascular smooth muscle cells (VSMCs) and pericytes, which surround the inner endothelial lining [1]. VSMCs circumferentially wrap around the inner layers of arteries, arterioles, veins and venules. The number of VSMC layers differs with the calibre and specification (venous or arterial) of the vessels. Pericytes are located in microvessels: capillaries, in which one or two ECs make up the inner perimeter of the blood vessel, precapillary arterioles and postcapillary venules [1], [2]. In larger vessels, fibroblasts and matrix form an additional outer layer [1], which also contains a microvascular system: the *vasa vasorum*. Moreover, bone marrow-derived circulating cells can adhere to and/or infiltrate into the different layers of blood vessels [1]. Several vascular and inflammatory cells are differently involved in the maintenance of the integrity of blood vessels, and in vascular remodelling following disease or injury. In post-natal life, the growth potential of large vessels is mostly limited to development of collaterals following the obstruction of a large vessel or a vascular shunt [3]. Classically speaking, the microvasculature network can grow through angiogenesis (*i.e.*, formation of small vessels from a pre-existing vascular structure) or vasculogenesis (defined as the *de novo* formation of blood vessels starting from stem cells). However, stem and progenitor cells are now known to contribute to both vasculogenesis and angiogenesis. For the former, they can differentiate into vascular cells which represent the building blocks of new vessels. For the latter, they can act in a paracrine manner (*i.e.*, releasing proangiogenic factors) to promote angiogenesis. The vascular biology community started to use the term “post-natal vasculogenesis” when evidence of the existence of “endothelial progenitor cells” able to integrate in blood vessel structure were proposed by Asahara and others [4], [5] in the late 90s. Today, this term is not commonly used and “angiogenesis” is widely employed to describe the post-natal growth of microvessels.

A healthy and functional vasculature is essential to ensure tissue perfusion. Congenital and acquired vascular diseases create the need for surgical anatomical repair, endarterectomy and by-pass or replacement of (portions of) large vessels. The possibility to use portions of autologous vessels is limited in some patients, due to extensive atherosclerosis disease, previous harvest or insufficient time to proceed with harvest (such after aneurysm rupture). Biological homografts, xenografts and prosthetic grafts are currently used in the operating theatre, but they present some limitations, especially as substitute for smaller vessels such as coronary arteries and the lower leg and foot arteries where limited run-off (distal flow rate) is a significant determinant of flow rate and long-term patency. Moreover, in the context of paediatric surgery, they have the major limitation of not being able to remodel and grow in synchrony with postnatal cardiothoracic development. Hence, VTE, based on the use of (bio) materials that can be combined with stem cells and other cellular and molecular products, is currently being investigated as a revolutionary option that would meet these clinical needs. Vascular pathology does not only relate to macrovascular disease. It also affects the microcirculation, which can worsen tissue ischemia induced by obstruction of large arteries. Moreover, there are clinical situations where microangiopathy can cause tissue ischemia independently of arterial occlusion. Diabetes mellitus is one of the main causes of microangiopathy. New therapeutic solutions that would enable improved blood perfusion by enhancing the microcirculation in ischemic tissues are the object of intense basic and clinical research. Gene- and cell-based therapeutic neovascularization has been developed for more than 20 years and is now ready to deliver clinical success. The field has been reviewed in many articles [6], [7], [8], [9] and we will not treat this broadly, limiting discussion to the potential impact of microRNA (miR) targeting on it.

For both VTE and therapeutic angiogenesis, optimally functional vascular cells are required and researchers are working to develop protocols that enable improved performance of fully differentiated cells and increase the production and quality of vascular cells from stem and progenitor cells. An obvious approach to improve cell quantity and quality is to manipulate the molecular pathways controlling the regulatory processes contributing to cell survival, growth and cell phenotype. MiRs have the capacity to modulate relevant molecular signalling through post-transcriptional inhibition of multiple target genes. Importantly, the expressional manipulation of relevant miRs has been shown to induce stem cell differentiation into vascular cell lineages [10] and promote angiogenesis (reviewed in [11]).

In this article, we discuss the clinical needs and state-of-the-art approaches for VTE and therapeutic angiogenesis; we summarize the most relevant information on miRs involved in vascular biology and stem cell differentiation to vascular lineages and we propose how miR targeting could be used to improve VTE and to promote therapeutic angiogenesis. We also discuss the remaining obstacles toward clinical translation.

## 2 The clinical needs for vascular tissue engineering and therapeutic angiogenesis

### 2.1 Congenital heart defects

Congenital heart disease (CHD) includes different anatomical defects in the thoracic vessels and/or in heart vessels and chambers. Overall, it affects approximately 1% of all live births, making it the most common birth defect worldwide. The most complicated CHD cases require surgical correction using prosthetic replacement grafts in the form of conduits and patches. However, these prosthetic materials lack growth potential. Consequently, as the infant grows over time, multiple operations are needed to remove the previous graft and implant a new one to fit the larger cardiovascular system. Improvements in cardiac surgery techniques and use of better prosthetic materials have led to a remarkable improvement in early survival rate with a parallel increase in the number of patients with adult CHD [12], [13]. It has been variably estimated in the United States that between 800,000 and 1 million CHD patients are now adults and many will require some form of vascular conduit or valve replacement due to failure of their previous grafts [14]. It is therefore necessary to develop new modalities for the treatment of neonatal vascular and cardiac defects to reduce or remove the need for multiple surgeries. From the above, it is understandable why CHD surgeons look at VTE with the hope of creating living scaffolds to be implanted as vascular conduits, valves or patches that are able to grow together with their paediatric patients.

### 2.2 Arterial aneurysms

Abdominal and thoracic artery aneurysms develop from the combined effect of luminal pressure and weakening of the arterial wall. The most common site for aneurysm formation is in the infra-renal abdominal aorta. In the United Kingdom, the prevalence of abdominal aortic aneurysm is as high as 1.5% in men aged over 65-years (http://aaa.screening.nhs.uk/statistics)[15]. The prevalence of thoracic aneurysms has been estimated of circa 1 per 10,000 people in the United States. However, the most lethal aneurysm site is in the thoracic aorta. In the United States, more than twice as many deaths are attributed to thoracic aortic dissections than to abdominal aortic aneurysms [16]. In fact, 95% of thoracic aneurysms present as an acute event (either as rupture or dissection) without previous symptoms [17], [18]. In many other patients, arterial aneurysms are incidental findings and are asymptomatic until rupture. These patients can be offered prophylactic vascular repair when their fitness and the size of the aneurysm shows the likely benefit from this intervention. The current options for repair are either open surgery with a prosthetic inlay/interposition graft or endovascular repair with radiological placement of a stent graft. This group of patients who harbour signals for a silent killer would greatly benefit from effective therapeutic options capable of preventing the progression of the aneurysmal dilatation before a more invasive intervention becomes the only possible treatment.

The embryology of the thoracic and abdominal aorta is different, as is the aetiology of the aneurysm formation [19]. The abdominal aortic pathology is a degenerative condition, related to age and traditional atherosclerotic risk factors. Pathologically, extracellular matrix destruction with VSMC apoptosis and increased inflammatory infiltration is observed. Thoracic aortic aneurysms have a stronger association with elastin degradation and VSMC proliferation [20]. At a more advanced stage of VTE development, the differences in the pathological features and the underpinning upstream regulatory pathways between these pathologies may have to be considered.

### 2.3 Ischemic disease

Ischemic disease in the heart and limbs is a main clinical need affecting large patient populations. In this clinical context, a combination of VTE for replacing the most severely affected or difficult-to-access-and-treat arterial vessels (usually those of smaller calibre) and stimulation of angiogenesis in the ischemic area could be synergic to improve clinical outcomes. MiRNA targeting could come in focus for improving both approaches.

Ischemic heart disease (IHD) is characterized by reduced blood supply to the heart caused by coronary artery disease (CAD), *i.e.* atherosclerosis in the coronary arteries. Diabetes mellitus (DM) heavily contributes to the prevalence and severity of IHD through aggravation of atherosclerosis and induction of microvascular disease [21]. Moreover, DM compromises the potential for native neovascularization responses to ischemia [21]. IHD is a leading cause of morbidity and mortality worldwide. IHD patients often qualify for revascularization by coronary artery bypass graft (CABG) surgery. Every year, around 28,000 CABG procedures are performed in the UK (15–20% in patients with DM) (from bluebook.scts.org -Blue Book Online-Society for Cardiothoracic Surgery). The vessels commonly used for by-pass are the internal thoracic artery (aka “internal mammary artery”) and the long saphenous vein. Unfortunately, in 10 to 20% of patients full revascularization is not always possible due to aggressive disease (calcification), small target vessels or diffuse distal vessel disease [22]. VTE could provide a new therapeutic hope for these “no option” patients.

VTE could be also a potential option in patients with end-stage peripheral arterial disease (PAD). PAD affects 1 in 5 of the population over 60 years of age (incidence in population estimate 50–100 per 100,000). Rest pain, ulceration or tissue necrosis define a situation when PAD has progressed to critical limb ischemia (CLI), which puts the patients at risk of losing their leg. Surgical bypass of the affected iliac or femoral artery are possible therapeutic options for these patients. Autologous veins that are more durable are preferred to prosthetic conduits in cases where bypass is performed below the knee level. Current state-of-the-art in peripheral vascular surgery is (when possible) the use of autologous veins taken from a leg (saphenous vein) or arm (cephalic or basilic veins). When autologous conduits are not available, synthetic grafts made or either gelatin coated Dacron or expanded PTFE can be used. However, the patency rates of synthetic grafts are inferior to autologous conduits [23]. Hence, the majority of these patients have delayed amputation due to failure of revascularization. New VTE protocols producing vascular conduits with a good patency profile would represent a significant improvement.

While revascularization (with either autologous naïve pieces of arteries or veins, prosthetic material or bioengineered vessels) focuses on restoring arterial blood flow, therapeutic angiogenesis seeks to improve the microcirculation by stimulating new blood vessel formation. Increasing numbers of “proof-of-concept” studies in small animal models of ischemia point to therapeutic angiogenesis as a way to improve myocardial and limb perfusion. Evidence from these studies fuelled the concept that molecular and cellular therapies able to stimulate angiogenesis could aid therapy optimization and even represent an alternative option for those ischemic patients who are not eligible for revascularization. Nevertheless, further work is needed to achieve the clinical success of therapeutic angiogenesis. In view of recent literature from our groups and others (reviewed in [24]), we propose that miR targeting could enable further progress in the translation of therapeutic angiogenesis to clinical practice.

## 3 Prosthetic materials clinically approved for large blood vessel repairs

The most common materials used for patch reconstruction of vascular and cardiac defects in neonates and infants born with CHD include various form of knitted polyester (most commonly Dacron), expanded polytetrafluoroethylene (such as Gore-Tex®), and several types of xenografts and homografts. The use of these vascular conduits is quite satisfactory in large diameter applications. However, they perform poorly in smaller vessel replacement. The blood-contacting surface and the biomechanical properties of the graft are believed to be important variables in causing patency in small vessels. Acellular or decellularised scaffolds have proved useful, as they provide the natural cues needed for recipient's cells to grow on the scaffold in terms of mechanics and composition [25] and it has been shown that the extracellular matrix (ECM) can still support cell growth even though many of its components have been disrupted [26], [27]. It is hard to remove 100% of cytoplasmic debris and nuclear materials from a biological scaffold and the consequence of remaining material in terms of host response remains unclear [28]. Early data indicated that decellularization of animal tissues is capable of producing stable vascular conduits that exhibit long-term functionality in other species [29]. However, the subsequent clinical experience has been disappointing [30], with severe degeneration of the grafts after only one year from implantation. Macroscopically these grafts showed severe inflammation and degeneration of the wall. Histology demonstrated severe foreign body type reaction; and significant calcific deposits were demonstrated [30]. No cell repopulation of the animal matrix occurred [30]. It is thought that the strong inflammatory response in humans may be due to matrix immunogenicity being conserved or may indicate insufficient decellularization to remove all xenoantigens [30]. Other forms of vascular grafts include decellularised tubular organs. Examples include porcine aorta, common carotid and iliac arteries [31], [32], [33]. Among gluteraldehyde-fixed xenografts, bovine and porcine pericardial patches have been widely used by CHD surgeons for vascular and intra-cardiac reconstruction. More recently, decellularised porcine small intestinal submucosa ECM has also been employed in the clinic [34], [35]. These biological materials have many technical merits, including easy handling and less suture bleeding compared with the synthetic patches. They are also greatly biocompatible, derived from a biological material that is fixed in glutaraldehyde, increasing the strength and stability of the material, while decreasing antigenicity and potential infections. However, these materials have also significant drawbacks, including limited ability to remodel or regenerate, the development of intimal hyperplasia, pseudo-aneurysm formation, restenosis (especially in small vessels), fibrosis and calcification that can lead to graft failure and the need for re-intervention by the surgeon. From all the above, it is clear that the “ideal” replacement vascular graft, which should be durable, biocompatible, non-immunogenic, non-thrombogenic, readily available, easy to handle, and have the potential to grow and remodel *in vivo*, does not yet exist.

## 4 Vascular tissue engineering

Tissue engineering (TE) involves seeding cells in three-dimensional matrices to form living tissue products having structural and functional properties that can be used to restore, maintain or improve tissue function. Success depends on the right combination of cells, a scaffold (either biologic or a synthetic material), and the necessary signals. TE approaches directed to building vascular conduits promise to overcome the limitations of the vascular grafts currently used in clinical applications. Moreover, the utilization of matrices already approved for clinical application could help to fast-track the translation of the tissue engineered product to patient's bedside.

Vascular ECs and mural cells (VSMCs and pericytes) are the essential components in vascular architecture and hence essential building blocks for vascular tissue engineering (VTE) [36]. These vascular cells can be obtained starting from differentiated blood vessels or through the differentiation of stem and progenitor cells. The latter process could be finalized before or after cells are seeded in the scaffold and even after scaffold implantation. The mechanical properties of the vessel are mainly driven by the VSMCs, which are also the numerically predominant cell population. In order to maintain vascular patency and sustain blood flow, the VSMCs should be in their non-proliferative and contractile status, which requires a correct interaction with the intra-luminal EC layer [37]. Moreover, a healthy and continuous endothelium layer ensures the right degree of interaction with and response to intra-luminal circulating cells and subcellular components, which is important in maintaining vascular integrity, avoiding vascular wall infiltration by inflammatory cells and intravascular thrombosis.

For any TE approach, ideally cells should be autologous or non-immunogenic and easy to acquire and culture in a clinical-grade manner. Table 1 summarizes the cell types previously employed by VTE scientists. ECM is essential for ensuring the mechanical properties that are critical to the function of the engineered blood vessel and hence it represents another essential ‘ingredient’ for VTE. The search for optimal sources of cells that are readily available, with shorter and easier culture times and good ECM production is still ongoing. A complete biological tissue engineered blood vessel was first produced by Weinberg and Bell using cultured mature VSMCs and ECs in bovine collagen gels [38]. Although this graft structurally mimicked a native artery, it was not functional due to very low burst pressure. From then on significant efforts have been put into engineering vascular grafts that mimic native vessels in composition and mechanical properties. The approaches that have been established to date still require long culture times and have shown varying degrees of success [39], [40], [41], [42]. Even so, even the most successful attempts are still unsatisfactory, failing to meet the criteria for use in clinical practice. Therefore, there is large scope for improving the process by acting at different steps. In this article, we focus on micro RNA (miR) targeting as a possibility to improve VTE, which includes acting at the level of cell production and on determining the type and quantity of growth factors (GFs) and ECM in the scaffold. Given the very short half-life of exogenously added GFs, it might be desirable to engineer the cells to increase their production in the scaffold [43]. Some miRs have been shown to regulate the expression of GF and their receptors in vascular cells [44], [45]. Hence, targeted miR therapeutics may provide an approach toward this goal. The ECM is mainly composed of fibronectin, laminin, collagens, elastin and hyaluronic acid and provides a structural framework to support cellular function and regulate cellular behaviour, including adhesion, proliferation, migration and differentiation of vascular cells through both cell-to-cell and cell-with-matrix interactions. Integrins and several proteases, especially matrix metalloproteinases (MMPs) regulate these processes, impacting on matrix remodelling and stiffness [43]. It is therefore conceivable that, as described below, modulating the expression of miRs to control ECM function in vascular cells or vascular progenitor cells before or after cell seeding into the scaffold might help to improve the properties of ECM and ultimately of the cell-engineered vascular graft.

## 5 microRNAs controlling vascular biology and the differentiation of stem cells to vascular lineages

### 5.1 MicroRNA biogenesis and mechanism of actions

MiRs are endogenous small non-coding (nc) RNAs. MiRs are encoded by autonomous miR genes or intronic sequences embedded in protein coding genes. A large proportion of miRs are clustered in polycistronic units. The canonical miR biogenesis for autonomous miR genes starts with RNA polymerase II-mediated transcription, producing a primary miR (pri-miR) of variable length. This is followed by pri-miR cleavage by a nuclear RNase complex, Drosha/DGCR8, generating a precursor miR (pre-miR) that has a hairpin-like secondary structure [46], [47]. Next, the pre-miR is translocated out of the nucleus to either the cytoplasm or the rough endoplasmic reticulum (ER) and subject to further enzymatic processing by the RNAse Dicer and its cofactors, producing a ≈ 22 nucleotide long double strand composed by two miR sequences [46], [48], [49], [50], [51]. One or both strands of the duplex can be recruited into the RNA-induced silencing complex (RISC), a RNA-protein complex that contains Dicer, other RNA binding proteins and Argonaute (Ago) proteins and that enables the miR to repress the expression of its “target genes” [52]. A miR recognizes its mRNA targets mostly through its “seed sequence” of 8 nucleotides, located in the miR 5′ untranslated region (5′-UTR) sequence which is (semi)complementary to one or more miR binding sites in the mRNA 3′-UTR. The end result of miR action is the repression of protein production from its targeted mRNAs. This can be reached through miR-induced mRNA degradation, transcript deadenylation, inhibition of translation, or sequestration of the mRNA in the processing body (P-body), where it is degraded or stored. The contribution of each mechanism remains debated [53], [54], [55], [56]. By regulating the expression of several target genes, each miR has the potential to imprint a strong molecular signature. Furthermore, individual mRNAs can be targeted by multiple miRs, allowing for high combinatorial complexity and regulatory potential. In fact, miRs are predicted to exert fine-tuning of posttranscriptional regulation to > 60% of mammalian protein-coding mRNAs [57].

**Fig. 1:**
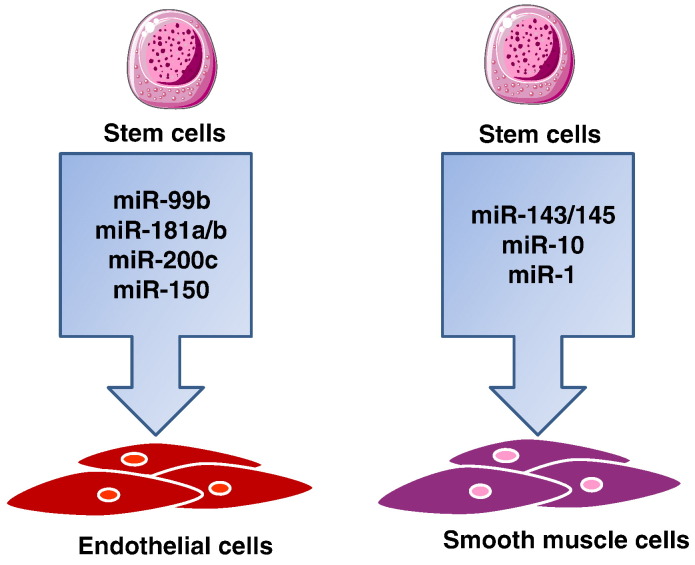
microRNAs reported to be enhanced on vascular differentiation of stem/progenitor cells. MiR-99b, -181a/b, -200c and -150 induce differentiation of human ESC to endothelial cells. miR-1 and -145 induce smooth muscle cell differentiation from human cardiomyocyte progenitor cells and human ESC respectively. miR-10 induces mouse ESC differentiation to smooth muscle cells.

In the aforementioned described clinical settings, therapeutic miR targeting could help to: 1) enhance the quantity and/or quality of cells available for cell-based therapeutic angiogenesis and/or for VTE; 2) promote stem cell differentiation to vascular cells to be seeded in the scaffolds (see Fig. 1); 3) improve the function of the cells in the vascular scaffold acting at different levels (see Table 2); and 4) correct anti-angiogenic molecular defect and/or amplify endogenous proangiogenic pathways directly in the ischemic tissue, thus promoting therapeutic angiogenesis leading to blood flow recovery. To reach these goals, a good understanding of the impact of different miRs that modulate vascular and stem cell biology is essential. Here we focus on how miRs could be utilised to manipulate EC and mural cells in order to enhance the outcome in VTE and to therapeutically induce angiogenesis in patients with either IHD or PAD leading to CLI.

### 5.2 MicroRNAs that regulate endothelial cell function and angiogenesis

ECs form the inner layer of any blood vessel ensuring vascular homeostasis; and have an essential role during developmental and post-natal angiogenesis. Pre- or post-implant vascularization of TE structures is essential for their engraftment. Moreover pre- or post-implant endothelization of the VTE lumen is mandatory to enable the grafted vascular conduit to mimic healthy blood vessels, where the endothelium contributes to regulation of vascular dilatation with no intimal hyperplasia, calcification, atherosclerosis and thrombosis. In VTE, EC growth and maintenance have been traditionally promoted using GFs such as VEGF and TGFβ [58]. After recent studies reporting on number of miRs as key *in vitro* and *in vivo* regulators of vascular EC function, proliferation and growth, it is sensible to suggest that *ad hoc* miR targeting could be applied alone or in combination with GFs to improve endothelial coverage and endothelial function in VTE. Initial evidence for a functional role of miRs in vascular ECs was provided by the observation that mice carrying a *Dicer* hypomorphic allele die prenatally with severely disrupted blood vessel formation accompanied with retarded expression of early endothelial markers [10]. Moreover, early studies showed that silencing of either Dicer or Drosha impaired the proangiogenic capacities of ECs, which could be restored by co-transfection of mimics of members of the miR-17–92 cluster, one of the most important vascular miR systems. [59], [60], [61] In early studies, Poliseno et al. performed a large scale analysis of miRs in human umbilical cord ECs and showed that 15 highly expressed miRs have the receptors of angiogenic GFs as their putative direct targets [62]. It is now well established that several miRs are important for endothelial development, physiology and for angiogenesis [62], [63], [64], [65]. Therefore, we speculate that during VTE it would be possible to intervene therapeutically by inhibiting pathogenic or anti-angiogenic miRs as well as by overexpressing miRs that elicit positive effects on the endothelium. Several miRs have been described to be expressed and functionally relevant in ECs. For a general overview, the readers can refer to previous review articles [24], [66], [67], [68], [69], [70]. Here, we will discuss miRs whose manipulation might be beneficial to maintain an integral and functional endothelium in VTE and those miR targeting for inducing therapeutic angiogenesis in IHD and CLI.

One of the major hurdles in using fully differentiated ECs in VTE is that after about 70 cell cycles, ECs can no longer divide thereby potentially affecting the functionality of the construct [71]. The limited replicative capacity of adult vascular cells is due in part to the progressive shortening of the telomeres over time. Ectopic expression of telomerase in somatic cells using a retroviral vector has been shown to reverse the telomere shortening; nonetheless this approach raises safety concerns since random incorporation of retroviral vectors may activate oncogenes [72]. Recent studies have shown that miRs play a vital role in controlling EC senescence and proliferation. In details, miR-217 overexpression was shown to promote endothelial senescence and miR-217 inhibition by transfecting with antagomir-217 ultimately reduced senescence and increased angiogenic activity in “old ECs” [73]. Moreover, miR-34a has been shown to promote senescence in different cells, including cardiovascular cells [74]. miR-34a over-expression significantly increased EC senescence and impeded angiogenesis [75]. By contrast, miR-34 inhibition by seed-targeting 8-mer locked nucleic acid (LNA)-modified antimiR (LNA-antimiR-34) promoted angiogenesis in mouse ischemic models [76], [77]. Thus, we speculate that the inhibition of miR-217 or miR-34a as well as other miRs which could be discovered to induce EC senescence could be useful to obtain a healthy endothelial layer in VTE.

An alternative approach to seeding ECs directly onto scaffold would be to recruit progenitor cells from the circulation. The immature cells would eventually differentiate into ECs on the lumen of the scaffold. This *in situ* VTE approach takes advantage of the regeneration potential of the host cells to regenerate a blood vessel *in situ* following vessel implantation. The advantage of this approach (which is treated in more details in the 5.4 section) would be that the graft preparation would need less extensive *in vitro* culture and manipulation).

Moreover, for VTE, miRs that are expressed in ECs and regulated by flow are particularly relevant. A number of flow sensitive miRs have been identified and named as mechano-miRs [69]. They include miR-10a, -19a, -23b, miR-21, -663, -92a (and possibly other members of the miR-17-92 cluster), miR-143/145, -101, -126, -712, -205, and miR-155 (reviewed in [69]). The key signalling pathways that are targeted by these mechano-miRs include cell cycle, inflammation, apoptosis, and nitric oxide signalling [69]. Many of these miRs were initially identified as flow sensitive *in vitro* and were later found to play a critical role in endothelial (dys)function, inflammation and atherosclerosis *in vivo* (reviewed in [69]). For these reasons, it might be worth to test whether *ex-vivo* manipulation of these individual miRs or their combination could improve the performance of the engineered vascular conduit preventing its stenosis and thrombosis once this is implanted in patients.

**Fig. 2:**
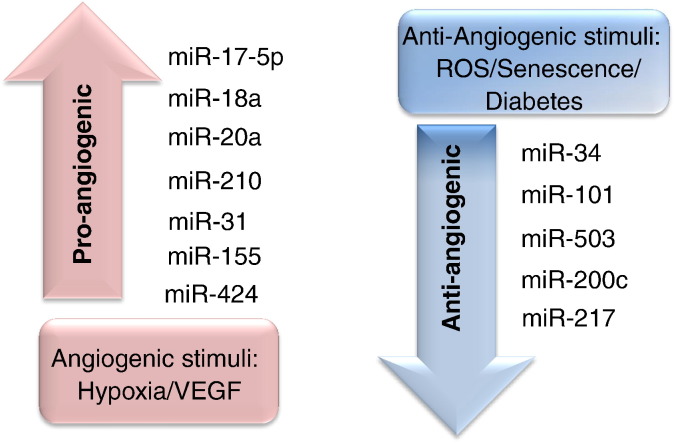
Angiogenesis regulatory miRs. MiRs that respond to pro-angiogenic stimuli or anti-angiogenic stimuli are enlisted.

Of importance for the therapeutic angiogenesis part of this article, the expression of miRs is regulated by hypoxia and ischemia [78], [79], [80], [81], [82], [83] and miRs shape post-ischaemic responses, including the vascular regenerative capacity of transplanted cells with proangiogenic capacity [11], [78] (Fig. 2). Based on the relationship between miRs and other angiogenesis regulators, we propose to classify angiogenesis-related miRs into two groups: 1) miRs that directly target genes regulating angiogenesis 2) miRs that can be modulated by pro-angiogenic or anti-angiogenic stimuli. The first group includes the so called ‘angio-miRs’, encompassing miR-126, miR-221/222 and the miR-17-92 cluster [66]. The second group of miRs can be controlled by either single molecules or more complex environmental conditions that promote or impair the angiogenesis process. For example, the Sessa laboratory found that VEGF-A induces time-dependent expression of miR17-5p, -31, -155, -18a, -20a in HUVECs [84]. Hypoxia was found to enhance the expression of two pro-angiogenic miRs, miR-210 [65] and -424 [85] in ECs. On the other hand, a number of miRs are regulated by anti-angiogenic signals such as ROS, senescence, high glucose *etc.* Study conducted by Magenta et al. showed that the negative effects of ROS on EC survival was due to the upregulation of miR-200c [86]. MiR-217 [87] and miR-34 [88] are reported to be upregulated in ageing ECs. Inhibition of these two miRs with specific antagomirs ultimately reduced senescence and increased angiogenic activity possibly *via* an increase in SIRT1 activity [73], [88]. The most studied miRs regulated by pro/anti-angiogenic stimuli are listed in Fig. 2. We have recently reviewed the topic of miRs in vascular context and we refer the readers to this previous publication [24].

Finally, diabetes mellitus has a severe damaging effect on the ECs in both large and small vessels [21], [89]. Part of these actions is mediated by miRs, including miR-503 [90] and miR-101 [91]. In two separate studies, we have reported increased miR-503 and-101 expression in EC exposed to type 1 diabetes mellitus and gestational diabetes, respectively [90], [91]. Moreover, forced overexpression of miR-503 and miR-101 in cultured ECs resulted in impaired cell migration, proliferation and network formation [90], [91]. When considering use of VTE and therapeutic angiogenesis in diabetic patients, it is sensible to consider if an additional pool of miRs should be targeted to improve the clinical outcome.

### 5.3 MicroRNAs that regulate pericyte function

As aforementioned, pericytes play important role in providing stability and nurturing signals to microvessels and maintaining proper capillary function, including in the *vasa vasorum* [92]. The use of pericytes in vascular regenerative medicine approaches has been limited by the lack of knowledge in the biology of these cells in comparison to the more widely studied ECs and VSMCs [93]. Moreover, there have been difficulties in isolating and expanding pericytes from human tissue samples. To harness the full potential of pericytes for VTE, the miR profile of these cells should be more carefully investigated. Murine microvascular pericytes have been shown to express miR-145 [94]. Moreover, a miR array performed on rat cortical pericytes has shown expression of several miRs and their regulation by hypoxia [95]. As described below, we were the first to identify miR-mediated actions in human pericytes [78].

At a more advanced stage of VTE optimisation, the engineered vessel should contain *vasa vasorum* (which include pericytes) to support its perfusion. Currently, the vascular architecture has not been engineered so precisely. However, the feasibility of using pericytes in VTE was shown in a recent study which used bi-layered elastomeric poly (ester-urethane) urea scaffolds [96]. Pericytes (3 × 10^6^ cells) were derived from human skeletal muscles and bulk seeded onto the scaffolds using a rotational vacuum seeding device in less than 2 min [96]. The seeded scaffolds were cultured in spinner flasks for 2 days and then implanted to immunocompetent Lewis rats as aortic interposition grafts for 8 weeks [96]. Pericytes successfully populated the porous layer of the scaffolds evenly after the dynamic culture and post implantation showed a significant higher patency rate than the unseeded control (100% vs 38%) [96]. In this study, the miR profile in the employed human cells was not analysed.

Recently, our group have succeeded in isolating and expanding a clonogenic population of pericyte progenitors (the “Bristol pericytes”) from saphenous vein left overs of patients undergoing coronary artery bypass graft surgery [97]. The Bristol pericytes have shown pro-angiogenic and anti-fibrinogenesis capacities when transplanted in mouse models of CLI [97] and IHD [78]. We have characterized the proangiogenic response induced by the Bristol pericytes *in vitro* using a co-culture system with ECs. Pericytes induced a paracrine activation of angiogenesis response by ECs through the release of miR-132 [78]. This response was augmented under hypoxic conditions, suggesting that this might be highly relevant in the proangiogenic actions induced by the Bristol pericytes once transplanted in the ischemic heart or limbs. In line with this hypothesis, complete *ex-vivo* inhibition of miR-132 expression with an antagomir in the pericytes before their transplantation in mice with IHD compromised the regenerative properties of these cells [78]. An added advantage of the Bristol pericytes is their low immunogenic profile and resistance to hypoxia/starvation [78]. MiR-132 additionally controls pericyte proliferation and survival *in vitro* [78].

From the above, overexpressing miR-132 levels in pericytes would improve their ability for reparative vascularisation. However, few miR overexpression strategies have been developed due to the caveat that miR mimic oligos are unstable, have transient effects and would require multiple administrations [24]. This could be overcome by using vessel targeted nanoparticles to systemically deliver the miR mimics directly to the site of post-ischemic angiogenesis and also in tissue engineered constructs. Anand et al. recently used vessel targeted nanoparticles to deliver antagomir-132 to block angiogenesis in a mouse tumour model [63]. A similar delivery approaches might be utilised for delivering miR-132 mimics for enhancing pericyte functions. Importantly, it was also observed that the expression levels of miR-132 in the Bristol pericytes can be upregulated up to 3-fold times by hypoxia or by stimulation with VEGF-B [78]. Hence it would be important to consider addition of factors such as VEGF-B and/or hypoxia to enhance miR-132 levels in the pericytes used for vascular regenerative approaches. We are currently moving our work with pericytes derived from adult and paediatric cardiac surgeries to TE approaches (Caputo, Angelini and Madeddu, unpublished data 2014 and 2015).

Ischemic diseases are shown to impair the pericyte recruitment to capillaries [98]. Hence supply of pericytes from exogenous sources appears therapeutically beneficial. An alternative to cell transplantation would be to target the pericytes resident in the ischemic heart and limb muscles with miRs that are able to stimulate their survival and function; as well as to inhibit miRs that have negative effects on these cells. To initiate this research, full genome miR profiling of human miR pericytes in IHD and CLI is fundamental; and this should be followed by expressional and functional validation efforts. In consideration of the fact that pericytes are severely compromised by diabetes [99], [100], the screening and validation studies should be separately conducted on samples from diabetic and non-diabetic patients.

Collectively, these studies suggest the promises of human pericyte populations in VTE and therapeutic angiogenesis and stimulate more research on these cells and their therapeutic use. Finally, the possibility to empower pericytes through miR targeting is emerging.

### 5.4 MicroRNAs that regulate vascular smooth muscle cell function

It is now well established that the smooth muscle layer of the blood vessel contributes an important role in ensuring vessel homeostasis and functions. In addition to their vascular physiological roles, VSMCs are also important players in the vascular remodelling processes observed under pathological conditions [101], [102]. VSMCs are one of the most plastic cells in the body being able to switch between a differentiated (contractile) state and a proliferative (synthetic) phenotype depending on the signals received [101]. Following an injury, VSMCs dedifferentiate to promote repair of vessels and once the injury is resolved, “healthy” VSMCs should return to contractile phenotype [101]. Although this modulation is critical for vascular repair, this plasticity sometimes contributes to the development and progression of vascular pathologies. One of the limitations of current VTE prosthesis is stenosis caused by excessive proliferation of VSMC, termed as intimal hyperplasia [103]. Thus, the modulation of VSMC between quiescent ‘contractile’ phenotype and proliferative ‘synthetic’ phenotype is crucial when designing VTE approaches.

VSMC function was recently found to be heavily influenced by miRs [37]. While some of the miRs promote VSMC proliferation, others stimulate differentiation. MiR-143 and -145 are among the most highly expressed miRs in VSMC and they are known as critical regulators in VSMC differentiation. Studies conducted in miR-143/-145-deficient mice revealed that VSMC requires these two miRs to switch from its contractile to synthetic function [104], [105]. The mechanism by which miR-145 regulate VSMC phenotype was defined by the elegant set of studies by Cordes et al. which showed that the miR-143/-145 cluster targets a network of transcription factors that promote differentiation and repress proliferation in VSMC [106]. The significance of miR-143/-145 in human vascular diseases is suggested by the observation that miR-143/-145 levels are decreased in patients with aortic aneurysm and CAD [107], [108]. A recent study revealed that extracellular vesicles (EVs) derived from KLF2-expressing ECs promote the contractile phenotype of co-cultured VSMCs by downregulating target genes of miR-143/145 [109]. The concept of cell-to-cell communication by transfer of miRs is discussed in detail toward the end of this review.

Another important candidate would be the miR-221/-222 cluster. These miRs were found to be expressed in normal arteries and significantly up-regulated after balloon angioplasty [110]. Furthermore, inhibition of miR-221/222 *in vivo* significantly reduced neointimal formation, demonstrating for the first time the potential of VSMC miRs as therapeutic targets for treatment of vascular disease [111]. MiR-221/-222 were downregulated in the balloon-injured rat carotid artery *via* 2′O-Methyl-modified antagomirs [111]. The miR inhibitor was pre-loaded into F-127 pluronic gel and applied locally to the adventitia around injured artery segments [111]. This method of delivery reduced any potential systemic side effects. A similar approach could be considered for improving VTE before or immediately after implantation.

Collectively these studies highlight the important role played by miRs in maintaining the phenotype of VSMC. Direct and/or indirect modulation of these miRs would be beneficial in maintaining the “optimal” phenotype of VSMC for VTE. Along with miRs, GFs and environmental cues also play important roles in VSMC phenotype modulation. For example, studies conducted in human primary pulmonary VSMCs showed that PDGF signalling is a potent inducer of the synthetic phenotype and propose that it acts *via* induction of miR-24 [112]. miR-221 is also reported as a modulator of the phenotypic change of human pulmonary VSMCs in response to PDGF signalling [113]. Thus, identifying the underlying pathways and cytokines involved in miR modulation should allow us to use them alongside miR targeting in VTE. However one must be careful about the side effects, since the same miR may have different responses under different conditions, and hence miR therapeutics can elicit off-target effects. For example, TGF-β and BMP signalling was reported to induce a contractile VSMC phenotype by increasing miR-21 [114]. However, hypoxia induced upregulation of miR-21 was found to induce the synthetic phenotype [114]. Hence more in-depth studies in this direction are required to understand the best miRs and/or GFs combinations that would be beneficial for VTE.

Similar to ECs, VSMCs can become senescent, which is detrimental for the VTE process. The limited replicative capacity of adult vascular cells is due in part to the progressive shortening of the telomeres with donor age [115]. This is particularly relevant when planning autologous VTE approaches in adult patient population, while it can be a minor issue for paediatric CHD surgical cases. A recent study has shown that miR-34a induces VSMC senescence by SIRT1 downregulation and promotes the expression of age-associated pro-inflammatory secretory factors leading to arterial dysfunction [116]. Increased miR-34a as well as decreased SIRT1 expression was also observed in replicative-senescent human aortic VSMC. miR-34a overexpression by transfecting with miR-34a mimic in proliferative human aortic smooth muscle cells induced cell cycle arrest [116]. miR-34a ectopic expression significantly increased the percentage of VSMCs in G0–G1 phase at 24 h post-transfection when compared with negative control [116]. From the above and the work on miR-34 in ECs described in above Section 5.2, we can suggest miR-34a inhibition to be worth being tested in VTE protocols.

### 5.5 MicroRNAs that modulate extracellular matrix production

In an effort to produce ‘off-the-shelf’ vascular grafts with an adequate ECM, Dahl et al. seeded human VSMC onto PGA scaffolds *in vitro* that were later decellularised and stored till the time of patient need [117]. These acellular human-based vascular grafts could be implanted directly into the patient or could be seeded with autologous ECs for small diameter vessels. Though the patient waiting time could be significantly reduced with this approach, the donor-to donor variation in the ability of VSMC to generate ECM *in vitro* remains a major challenge. VSMCs are the major producers of ECM. The successful generation of tissue engineered blood vessels *in vitro* has been estimated to require almost 8–24 weeks of cell culture time to generate adequate ECM necessary for arterial grafts [39], [41]. The mechanical strength of the vessel is largely derived from the ECM components such as collagen and elastin. Older cells deposit less ECM than younger cells due to downregulation of ECM production and elastogenesis [118]. This leads to the generation of vessel grafts with poor mechanical properties, such as burst pressure and suture retention [118]. A recent study has shown that inhibition of miR-29a can dramatically increase elastin expression in human cells and that miR-29 inhibition upregulates elastin levels in cells from patients with elastin haploinsufficiencies and in bioengineered human blood vessels [115]. In this study, polyglycolicacid (PGA) scaffolds were seeded with early passaged human VSMCs and the vessels were grown in a bioreactor for 6 weeks in the absence or presence of miR-29a inhibitor. Complete miR-29a inhibition increased the appearance of elastin “islands”, thereby increasing the distensibility of the bioengineered vessels at low pressures [115]. Thus, antagonizing the actions of miR-29 may promote increased elastin levels during conditions of enhanced elastinolysis or deficiencies.

### 5.6 MicroRNA targeting for improving the process of stem cell differentiation to vascular cells and the functional capacities of vascular progenitor cells

The re-population of a decellularised matrix with a patient's own stem cells can potentially create a living structure with subsequent long-term preservation of mechanical and biological properties. A number of studies have focused on techniques to promote vascular differentiation from stem cells. With the discovery of miRs, a new route to enhance stem cell differentiation to vascular lineages has presented itself. The possibility to achieve efficient generation of unlimited supplies of vascular cells and vascular progenitor cells from stem cells would be highly beneficial for VTE and therapeutic angiogenesis. Our group and others have established *in vitro* protocols for human embryonic stem cells (ESCs) differentiation to endothelial progenitor cells and ECs [119], [120], [121]. Microarray profiling of miR expression at subsequent stages of differentiation identified miR-99b, -181a and -181b as key miRs involved in the process [119]. Lentiviral mediated overexpression of these miRs in ESCs enhanced the efficiency of EC lineage commitment. Moreover the ESC-derived ECs showed improved capacity to promote post-ischemic blood recovery in immunocompromised mice with experimentally induced limb ischemia [119]. The findings from the study by Luo et al. suggest that as human ES cells differentiate to the EC lineage, miR-200c and miR-150 are upregulated resulting in EC differentiation [122]. The study by using *in vivo* inhibition of the mentioned miRs (using antagomirs), also showed that miR-200c and -150 played an important role in chick embryonic blood vessel formation [122].

Another study showed miR-126 to be abundantly expressed in mesodermal progenitor cells derived from stem cells and to regulate mature EC function. However, lack of an embryonic lethal phenotype with genetic miR-126 deletion suggests that miR-126 does not command early endothelial lineage commitment and it becomes relevant later during vascular differentiation [123]. Similarly the miR-17–92 cluster has been reported to be expressionally regulated during EC differentiation from pluripotent stem cells, but it was not shown to functionally impact on the process [124].

Dar et al. recently showed successful generation of pericytes from human ESCs and induced puripotent stem cells (iPSCs) [125]. Co-implantation of iPSC-derived ECs and pericytes into immunodeficient mice resulted in significant vascular and muscle regeneration [125]. Whether miRs contribute into stem cell differentiation into pericytes has not been clarified, yet. Therefore further studies are required.

MiR-143 and -145 are very well characterized in the vasculature and are positioned as a central target of multiple growth factor signalling pathways influencing VSMC differentiation and maintenance. A recent report has shown that overexpression of miR-145 can regulate the fate and phenotype of human ESC-derived pre-VSMCs as they fully differentiated to VSMCs [126]. Overexpression of miR-145 was induced by the transfection of 100 nM precursor miR-145 (premiR-145), which resulted in a greater than 3000 fold increase in miR-145 level in human ESC-pre-SMC. Overexpression of miR-145 repressed the expression of KLF4 (one of the pluripotency factors present in the “Yamanaka cocktail” used to reprogramme differentiated cells to pluripotency) [127]. It also upregulated the expression of smooth muscle markers, and changed the cell morphology into a differentiated spindle-like shape phenotype. Furthermore, introduction of miR-145 inhibited the proliferation of the ESC-pre-VSMC and increased their carbachol-stimulated contraction. In contrast, downregulation of miR-145 (knockdown by 100 nM miR-145 inhibitor — “antimiR-145”) in ES-pre-VSMCs upregulated KLF-4 and -5 expression and suppressed the expression of VSMC markers [126]. Collectively, these studies show that miR-145 can regulate the fate and phenotype of human ESC–VSMCs as they become fully differentiated VSMCs. Hence, miR-145 could be used to modulate stem cell differentiation to VSMCs for VTE. However, it should be noted that animals with miR-145 knock out are viable through adulthood, suggesting the role of additional miRs in the process of VSMC differentiation.

MiR-1 expression is reported to be increased during differentiation of mouse ESCs to VSMCs [128]. KLF4 was found to be a common target for both miR-143/145 and miR-1 [128]. The study showed that when miR-1 is overexpressed by transient transfection of miR mimic, KLF4 is repressed and VSMC differentiation is promoted [128]. MiR-1 is also reported to mediate the vascular differentiation of human cardiomyocyte progenitor cells, a cell population which can be derived from human atrial and ventricular tissue obtained as surgical waste and that can be differentiated into both VSMCs and ECs [129]. Another miR reported to be involved in VSMC differentiation is miR-10a [130]. The expression of miR-10 was found to be increased during mouse ESC differentiation to VSMCs [130]. Inhibition of miR-10 (transient knock down using miR inhibitor) resulted in a substantial reduction in VSMC differentiation [130]. Collectively, these studies provide the first evidence that miRs play a major role in directing and regulating stem cell differentiation into vascular cell lineages and hence that miR targeting may represent a valuable tool to improve the availability of vascular cells to be used in TE. Even if this approach has not yet been explored in VTE, a proof of concept was recently provided in bone tissue engineering. Mariners and team transfected human mesenchymal stem cells with mimics of miR-148-b and inhibitors of miR-489, two miRs that were linked to osteogenesis [131]. The investigators seeded these cells into osteoinductive scaffolds, which resulted in better ECM deposition and bone formation. This work on bone tissue engineering can potentially be used as a template for using miR modulation strategies for vessel regeneration.

MiR targeting could be additionally used to empower patient-derived autologous progenitor cells and circulating proangiogenic cells to be employed both in VTE and for direct transplantation into ischemic tissue. Moreover, we suggest that miR targeting approaches might improve the release of endothelial chemoattractant messengers from VSMCs seeded in scaffolds, which should facilitate the endothelization of the graft after its implantation by promoting EC migration from the bordering native vasculature as well as by recruiting blood circulating endothelial progenitor cells. Bone marrow (BM)-derived proangiogenic cells (PACs), which were previously known as early endothelial progenitor cells (EPCs), have been implicated in both native and therapeutically guided angiogenesis [132]. Our group have recently shown that miR-15a and miR-16 profoundly inhibit the migratory potential of PACs isolated from patients with CLI [83]. *Ex vivo* manipulation of PACs with anti-miR-15a/16 improved survival and migratory capacity of these cells and increased post-ischemic recovery of mice with limb ischemia [83]. Moreover, Tano et al. showed that the number of circulating mononuclear cells (MNCs) as well as of CXCR4 positive MNCs were increased by acute IHD (an heart attack) in patients and identified miR-150 as a key regulator for mobilizing these cells from the BM by targeting CXCR4 [133]. Knock down of miR-150 by transfection with its inhibitor in MNCs resulted in enhanced migration capacity. Moreover, lenti-virus mediated knock down of miR-150 significantly increased CXCR4 expression in MNCs and increased MNC mobilization from BM to PB when transplanted into the BM of wild type mice [133]. This indicates that miR-150 is associated with stem cell mobilization by targeting CXCR4. Another study showed that microvesicle based exogenous miR-150 delivery to human microvascular EC enhanced their migration ability *in vitro* [134]. Collectively these studies show that miRs influence recruitment of progenitor cells and could be used as a therapeutic tool for effective host cell mobilization to the lumen of the scaffold in VTE.

## 6 MicroRNA therapeutics

As discussed earlier in this review, miRs have an essential role in vascular biology and pathology as well as in stem cells differentiation into vascular cells. To fully utilise the power of these master regulators in VTE, there are several aspects that needs to be answered effectively. Firstly, the choice of miR modulation strategies needed to give the desired effect in VTE should be carefully considered. Secondly, how these strategies would be delivered/used in a VTE perspective. We will look into the approaches for therapeutic miR delivery strategies in the following section.

Studies conducted over the last decade clearly show that miRs are involved in almost all types of cellular processes, making them an attractive therapeutic target that could be used in VTE. The clinical studies conducted so far using GFs to enhance or modulate the cell source in VTE has met with limited success. The limitations of current techniques could be effectively overcome by the adequate use of “miR drugs”. Strategies for miR-based therapies are based on either the restoration of suppressed genes by inhibiting the specific miR and or the delivery/overexpression of the miRs that suppress those target genes that have a desired effect on the cell source or signal used in VTE. However, care must be taken when targeting miRs that have multiple targets to avoid unexpected side effects. MiRs are differently involved in the maintenance of a variety of cell types. Therefore it cannot be excluded that the same miR can be advantageous to the cell population we want to target, but have negative side effects on other cells or tissues. Hence, it becomes evident that a finely tuned “miR drug” delivery is needed to avoid potential harmful side effects. For example, the aforementioned miR-34a involved in vascular cell senescence [75], [116], is one of the most characterized tumour suppressor miRs in a variety of tumours [135]. Based on these considerations, chemical modifications aimed to improve the biological stability of selected miRs could be successfully combined with targeted active delivery approaches, allowing easier clinical translation.

### 6.1 “MicroRNA drugs” delivery during the vascular tissue engineering process

Selecting the right miR drug delivery strategy for VTE is crucial in determining the success of the construct. The major challenges in delivering plasmid and oligonucleotide-based miR therapies are nuclease degradation, limited cell membrane permeability, and generally transient activity. Delivery strategies must be selected based on the class of miR therapy chosen and also on the target tissue. For instance, for localized applications, such as vascularising a tissue engineered construct that can be easily accessed, a direct tissue injection can be optimal. Controlled release of miRs could be achieved by using biodegradable scaffolds that can simultaneously act as a tissue template and as a source of sustained miR release [136]. This would also allow more spatiotemporal control over miR activity. It would be also ideal to do material surface immobilisation of “miR drugs” to enhance local delivery and to minimize off-target effects. In cases where the tissue is not easily accessible, systemic delivery approaches should be considered [90]. The delivery of synthetic oligonucleotides in NPs or microparticles could be used to prevent their rapid degradation by cellular and serum nucleases and could prevent them from crossing through cell membranes. This could significantly reduce off target effects and ensure maximum therapeutic benefit. Two recent studies have successfully used NPs to deliver miRs able to modulate angiogenesis. Anand et al. used vessel targeted NPs to deliver miR-132 [63]. The study used an integrin αvβ3-targeted nanoparticle that can deliver nucleic acids or drugs to the tumour neovasculature for selective delivery of anti-miR-132 to the cancer endothelium in mice. Systemic administration of anti-miR-132 NPs blocked cancer angiogenesis [63]. The NPs were composed of distearoylphosphatidylcholine (DSPC), cholesterol, dioleoylphosphatidylethanolamine (DOPE), distearoylphosphatidylethanolamine (DSPE)-mPEG2000, and DSPE-cyclic RGDfK. Mice were treated with 50 μg of scrambled anti-miRNA or anti-miR-132-NPs intravenously every 2 days starting from day 12. Injection of a single dose of anti-miR-132 in the RGD NPs decreased tumour size transiently with a half-life of biological efficacy of 2 days [63]. These NPs can be used to deliver inhibitor of miRs that impair angiogenesis and the VTE process. They could also be employed to deliver mimics of miRs that are beneficial in this context. In summary, this study suggests a good approach for targeted NP-based miR delivery that could be utilised with VTE.

Another approach would be to *ex-vivo* deliver miR therapy to cells to modulate cell differentiation or survival as required for the VTE construct. In a recent report Devalliere et al. report on a new approach for enhanced EC transplantation using targeted NPs transfection to deliver proangiogenic miR-132 to cultured ECs before their transplantation, thereby sensitizing cells to the effects of endogenous GFs [137]. Transfection of ECs with miR-132 enhanced GF-induced proliferation and migration in 2D culture [137]. However, while the effects of conventional transfection were short-lived, nanoparticle transfection produced protein knockdown and biological effects that were significantly longer in duration (≥ 6 days) [137]. Transfection of ECs with miR-132 NPs resulted in a 2-fold increase in the number of microvessels per square millimeter compared to lipid after transplantation into immunodeficient mice and led to a higher number of mural cell-invested vessels than control transfection [137]. These data suggest that it would be possible to modulate cell characteristics using miR therapy prior to their use in VTE.

### 6.2 MicroRNA delivery *via* extracellular vesicles

Functionally competent mature miRs are released by cells as conjugates with (lipo)proteins [138], [139], [140], and/or loaded in extracellular vesicles (EVs, exosomes), microparticles and apoptotic bodies [139], [141]. Being inside EVs or conjugated to (lipo)proteins makes extracellular miRs resistant to degradation [142]. Exosomes and microparticles (MPs) can transfer biologically active miRs to neighbouring or distant cells [143]. MiR exchange *via* exosomes has been shown to modulate cross-talk between ECs and VSMCs [109] and pericyte with ECs [78]. It is important to note that the functionality of EVs can be enhanced by modulating the culture conditions of the producing cells. For example Lopatina et al. showed that the functionality and number of adipose MSC-derived EVs can be increased by PDGF stimulation [144]. Another study by Zhang et al. showed that hypoxic stimulation was required to obtain functional EVs from BM–MSC [145]. Collectively, these studies suggest that EVs with the ‘right’ miR or miR combinations can be used directly to deliver therapeutic miRs to ischemic tissues and at different steps of the vascular TE process. After isolation, EVs can be utilised either separately or in combination with cells or other therapeutics for VTE. Direct injection could be one way of EV delivery into the tissue or circulation. It can be speculated that EVs can be mixed with scaffolds during preparation or coated onto the scaffolds *via* chemical linkers, antibodies or by specific tags. Use of EVs with biodegradable scaffolds with desired release profiles would allow constant and gradual release of EVs for the desired effect. A major advantage is that EVs would allow harnessing the paracrine effects of stem and progenitor cells without having to administer live (and potentially harmful) stem cell populations. This would help in easier clinical translation with regard to safety, regulation and complexity. However a major challenge that needs to be addressed is how to isolate the required quantity of EV for VTE. The current gold standard in isolation relies on ultracentrifugation [146]. Although commercial reagents are available for higher yields, these products still require a lot of optimisation [146]. As an alternative, artificial EVs can be engineered with the desired miR cargo to be delivered during VTE and for therapeutic angiogenesis in IHD and CLI. However, more research is required in this direction to confirm the feasibility of this approach. The optimization of EV production would allow one to regulate the cargo release in response to different physical parameters, including pH, temperature *etc.* Moreover, the EV membrane could be engineered to contain epitopes guiding targeting and delivery into specific cell types. From the above, it is apparent that more studies are required to optimize the angiogenic potential of EVs for their effective use in VTE and therapeutic angiogenesis.

### 6.3 Open questions and key challenges in microRNA therapeutics for vascular tissue engineering

The studies discussed here indicate that miR therapeutics can have wide applications for VTE supporting the enthusiasm for further exploration of miRs as novel therapeutic candidates. However numerous challenges remain in the path toward clinical translation of miR therapeutics for VTE. miRs are master regulators targeting several regulatory networks at the same time. Though this can be highly advantageous in certain scenarios, the promiscuity of miRs also makes the process of selecting the right therapeutic miR candidates very challenging. There is inevitable overlap between key genes controlling pluripotency and differentiation on the one hand cancer initiation and development on the other [147]. A clear understanding of the complex regulatory networks by bioinformatics, systems biology and target prediction algorithms is essential. In most of the animal studies to date, the phenotypic effects of miR modulation have only been studied in the target tissue of interest, which might have overlooked off-target effects in additional tissues. Moreover, the doses used in most pre-clinical studies are unlikely to be clinically feasible. It would be important to identify optimum dosing regimens to establish the lowest possible efficacious doses without side effects.

Another important factor that needs to be considered is that tissue repair by VTE is a multi-step process that requires variable levels of miR modulation depending on the stage of the TE process. Identifying the miRs that are modulated during biological vascular repair in the body would be very important for VTE. The best outcomes in miR therapy for VTE might occur when the temporal provision of miR modulators are matched to these biological repair processes. MiR modulation strategies are based on the presumption that relatively modest changes in the expression of target mRNAs are sufficient to evoke therapeutic benefits. An interesting study by Mukherji et al. showed that mRNA targets by miRs display threshold effects such that the mRNA can be efficiently modulated when it is present at relatively low levels compared to miR [148]. However, when the mRNA is present at a far higher level, the biological impact of miR becomes diminished since the miRs will not be able to inhibit it anymore [148]. Hence swamping a system with miR overexpression or inhibition strategies may be insufficient to achieve the desired outcome. Another study has shown that transfecting with miR mimics and miR inhibitors affects the expression of genes predicted to be under endogenous miR regulation [149]. This effect was observable at both the mRNA and protein levels [150]. Apart from controlling the miR levels, precision of targeting is also essential to ensure that the modulators are delivered to the targeted cell population. In summary, for miR therapeutics for VTE needs intelligent target selection coupled with precise spatiotemporal modulation of miR targets. This is not a very easy task and requires extensive research.

## 7 Conclusions and future perspectives

Development of a tissue engineered vessel that is mechanically robust, biologically functional and clinically compatible is extremely challenging. From a clinical perspective, time and cost are important factors that need to be considered for VTE. The most successful approaches so far have utilised vascular cells and the process still require long times for vessel generation. MiR modulation of cells for use in VTE is an exciting, underexplored avenue. As discussed in this review, a number of miRs have been reported to control vascular cell and stem cell properties that determine vital therapeutic outcomes in VTE. It may be possible to combine several modulatory strategies to harness the ‘right’ miR expression signature for a desired clinical benefit. miR expression levels in vascular cells or progenitor cells could be manipulated *in vitro* using either an inducible or suicide cassette. This would allow constitutive expression of miRs and stringent governance of temporal expression to target dysfunctional gene expression. This may also open up new alternatives for obtaining autologous cells in a more time efficient manner for VTE. However, it is important to be careful with implementing the exciting miR targeting technologies because a complete understanding of all the processes is still lacking. As a consequence, miRs have not yet reached clinical application. From a translational point of view, *ex-vivo* use of miR targeting for improving the production and functional properties of cells to be either used for therapeutic angiogenesis approaches *via* direct transplantation into ischemic tissue or as building blocks for bioengineered scaffolds would improve the risk profile of any “miR drug”. This would also facilitate the entrance into the clinical arena *via* a first-in-man clinical trial, which is now enthusiastically anticipated by the whole community of basic and clinical scientists whose attention has been captured by miRs.

## Sources of funding

BHF Centres of Regenerative Medicine (CE); Leducq Transatlantic Network in Vascular microRNA (MIRVAD) (CE); Sir Jules Thorn award 2014 (MC); the National Institute for Health Research (NIHR) through the Bristol Biomedical Research Unit (BRU) in Cardiovascular Medicine (GDA). The views expressed are those of the author(s) and not necessarily those of the National Health System, the NIHR or the Department of Health.

## Figures and Tables

**Table 1:** Cell types employed in the vascular tissue engineering approaches trialled in the clinics.

Cell type and source	Application	Preparation time	Follow-up time	Patency	Reference
Autologous ECs derived from the external jugular vein and cephalic vein	Saphenous vein graft	2 months	3 years	27/33	[151]
Autologous vascular cells derived from peripheral vein biopsies	Pulmonary artery	2.5 months	7 months	3/3	[152]
Autologous BM-MNC derived from iliac crest	EC-TCPC	Hours	7 years	25/25	[42]
Autologous fibroblasts and endothelial cells isolated from skin and vein biopsies	Arteriovenous us shunt	4 months	13 months	6/6	[41]
Autologous bone marrow stem cell-derived EC and VSMC	Extra-hepatic portal vein	1 month	1 year	1/1	[40]

BM-MNC: bone marrow mononuclear cells; EC: endothelial cells; EC-TCPC: extracorporeal total cavopulmonary connection; VSMCs: vascular smooth muscle cells.

**Table 2:** Possible miR targets for vascular tissue engineering dedicated to patients.

miR	Target	Targeted cells	Modulation	Modulation strategy/factor	Outcome	Ref
17–92	Jak1	EC	Downregulation	Antagomir	Increased angiogenesis	[153]
217	SIRT1	EC	Downregulation	Antagomir	Reduced senescence and increased angiogenesis	[73]
34	Vinculi, Notch1, semaphorin 4B	EC	Downregulation	LNA	Increased angiogenesis	[76]
143/145	Klf-2	EC, VSMC	Upregulation	Laminar shear stress	Inhibit VSMC dedifferentiation	[109]
19a	Cyclin D1	EC	Upregulation	Laminar shear stress	Induce endothelial senescence	[154]
155	MYLK	EC	Upregulation	Laminar shear stress	Inhibit EC proliferation and migration	[155]
92a	Klf-2/4	EC	Upregulation	Low shear stress	Induce endothelial inflammation	[156]
663	Klf-4	EC	Upregulation	Oscillatory shear stress	Induce endothelial inflammation	[157]
126-5p	Dlk-1	EC	Downregulation	Disturbed flow and low shear stress	Inhibit EC proliferation	[158]
10a	MAP3K7	EC	Downregulation	Disturbed flow and low shear stress	Induce EC inflammation, hyperpermeability	[159]
205/712	TIMP3	VSMC	Upregulation	Disturbed flow	Induce VSMC migration	[160]
126	SPRED-1, PIK3R2	EC	Upregulation	Blood flow	Pro-angiogenic	[161]
17-5p	TIMP1	HUVEC	Upregulation	VEGF	Pro-angiogenic	[162]
18a	Tsp1	HUVEC	Upregulation	VEGF	Pro-angiogenic	[162]
31	UD	HUVEC	Upregulation	VEGF	Pro-angiogenic	[162]
155	ATR1	EC	Upregulation	VEGF	Pro-angiogenic	[162]
210	Ephrin A3	EC	Upregulation	Hypoxia	Pro-angiogenic	[65]
424	CUL2	EC	Upregulation	Hypoxia	Pro-angiogenic	[85]
200c	Zeb1	EC	Upregulation	ROS	Induce EC death and senescence	[86]
217	SirT1	EC	Downregulation	Antagomir	Reduce senescence in ageing EC	[73]
34	SirT1	EC	Downregulation	Antagomir	Reduce senescence in ageing EC	[88]
503	CCNE1, cdc25A	EC	Downregulation	Adenovirus-mediated miR-503 decoy delivery	Enhance post LI vascular repair	[90]
101	EZH2	HUVEC	Downregulation	Antagomir	Increased EC tube formation and migration	[91]
132	RasGTPase activating protein, methyl-CpG-binding protein 2	Pericytes	Downregulation	Antagomir	Decreased pericyte capacity to improve contractility, reparative angiogenesis	[78]
143/145	Elk-1, Klf-4	VSMC	Downregulation	Antagomir	Induction of synthetic phenotype	[106]
221/222	p27 (Kip1), p57 (Kip2)	VSMC	Downregulation	2′-O-methyl modified antagomir	Induction of contractile phenotype	[110]
21	Pten, Bcl2	VSMC	Upregulation	TGF-b and BMP signalling	Induction of contractile phenotype	[114]
21	PDCD4, Sprouty-2, PPAR	VSMC	Upregulation	Hypoxia	Induction of synthetic phenotype	[114]
24	Trb3	VSMC	Upregulation	PDGF β	Induction of synthetic phenotype	[112]
34a	SIRT1	VSMC	Upregulation	miR-34a mimic	Induction of cell cycle arrest	[116]
29a	VDAC1/2	VSMC, fibroblasts	Downregulation	miR-29a inhibitor	Induce elastin production	[115]
99b, 181a, 181b	Prox1	hESC	Upregulation	Lentiviral	Enhance efficiency of EC lineage commitment, improved post ischemic blood flow recovery	[11]
200c, 150	Zeb1	hESC	Upregulation	Precursor miR-200c, 150	Induce differentiation to EC	[122]
145	KLF-4/5	hESC	Upregulation	Precursor miR-145	Induce differentiation to VSMC	[126]
1	KLF-4	mESC	Upregulation	miR-1 mimic	Induce differentiation to VSMC	[128]
10a	histone deacetylase 4	mESC	Downregulation	Inhibitor	Reduction in VSMC differentiation	[130]
15a/16	VEGF-A, AKT-3	EPC	Downregulation	Antagomir	Improved survival and migratory capacity	[83]
150	CXCR4	MNC	Downregulation	Lentiviral	Increased MNC migration from bone marrow to peripheral blood	[133]

ECs: endothelial cells, VSMCs: vascular smooth muscle cells, HUVECs: human umbilical vein endothelial cells, hESCs: human embryonic stem cells, mESCs: mouse embryonic stem cells, EPCs: endothelial progenitor cells, MNC: mononuclear cell, UD: undefined.
